# Latent Profile Analysis of Schizotypy and Paranormal Belief: Associations with Probabilistic Reasoning Performance

**DOI:** 10.3389/fpsyg.2018.00035

**Published:** 2018-01-26

**Authors:** Andrew Denovan, Neil Dagnall, Kenneth Drinkwater, Andrew Parker

**Affiliations:** Department of Psychology, Manchester Metropolitan University, Manchester, United Kingdom

**Keywords:** framing effects, latent profile analysis, paranormal belief, probabilistic reasoning, schizotypy

## Abstract

This study assessed the extent to which within-individual variation in schizotypy and paranormal belief influenced performance on probabilistic reasoning tasks. A convenience sample of 725 non-clinical adults completed measures assessing schizotypy (Oxford-Liverpool Inventory of Feelings and Experiences; O-Life brief), belief in the paranormal (Revised Paranormal Belief Scale; RPBS) and probabilistic reasoning (perception of randomness, conjunction fallacy, paranormal perception of randomness, and paranormal conjunction fallacy). Latent profile analysis (LPA) identified four distinct groups: class 1, low schizotypy and low paranormal belief (43.9% of sample); class 2, moderate schizotypy and moderate paranormal belief (18.2%); class 3, moderate schizotypy (high cognitive disorganization) and low paranormal belief (29%); and class 4, moderate schizotypy and high paranormal belief (8.9%). Identification of homogeneous classes provided a nuanced understanding of the relative contribution of schizotypy and paranormal belief to differences in probabilistic reasoning performance. Multivariate analysis of covariance revealed that groups with lower levels of paranormal belief (classes 1 and 3) performed significantly better on perception of randomness, but not conjunction problems. Schizotypy had only a negligible effect on performance. Further analysis indicated that framing perception of randomness and conjunction problems in a paranormal context facilitated performance for all groups but class 4.

## Introduction

Previous research has established that schizotypy and belief in the paranormal predict propensity to heuristic bias (see Dagnall et al., 2016b). Heuristics are simple shortcuts, or mental rules of thumb, that guide subjective estimation of event probability in situations of uncertainty (Gigerenzer and Gaissmaier, 2011). People use heuristics when likelihoods are unknown and/or information is complex or incomplete (Tversky and Kahneman, 1974, 1993). Such features are often evident in real-world situations, hence heuristics regularly guide everyday decision-making (Gigerenzer and Gaissmaier, 2011).

Cognitively, heuristic based decision-making is frugal and expedient. Since heuristics consider only limited evidence, they reduce cognitive load and facilitate rapid formation of judgments (Tversky and Kahneman, 1974; Shah and Oppenheimer, 2008). Acknowledging these characteristics, the predominant academic view of heuristic based decision-making is that it typically yields reasonable outcomes, but can on occasion produce severe and systematic error (Kahneman and Tversky, 1973). Although, theorists often link heuristics to sub-optimal judgments (deviations from probability, irrationality, and error-proneness), heuristics actually frequently produce accurate or adequate judgments (Costello and Mathison, 2014).

Germane to the current study was the notion that an association exists between heuristic judgments and illusory causation, specifically appreciation of chance (randomness). Illustratively, Dagnall et al. (2016b) found that misperception of chance (faulty perception of randomness) and conjunction error (fallacy) were associated with higher levels of schizotypy and belief in the paranormal. These biases explicitly index insensitivity to probability (Arnott, 1998, 2006).

Misperception of randomness denotes the tendency to perceive patterns and relationships within unconnected data/stimuli (Dagnall et al., 2007). This may manifest as the inability to judge accurately the likelihood of strings and sequences (Blackmore and Trościanko, 1985). With reference to belief in the paranormal, misrepresentation of randomness often expresses as illusory causation (magical ideation) (Dagnall et al., 2016b). Magical ideation is the propensity to infer causal relationships between unrelated events (Eckblad and Chapman, 1983). Preceding studies indicate that magical ideation is associated with higher levels of both schizotypy (Eckblad and Chapman, 1983) and paranormal belief (Williams and Irwin, 1991; Thalbourne and French, 1995).

Acknowledging this, Dagnall et al. (2016b) proposed that believers in the paranormal attribute/ascribe unwarranted connections to non-related stimuli in a belief consistent manner. For example, if whilst thinking about someone who has not been in contact for some time that person suddenly telephone calls or emails (coincidence), a believer in extrasensory perception (ESP) might view this occurrence as evidence for the existence of psi phenomena (such as psychic communication/telepathy or premonition/precognition) (Irwin et al., 2013).

Conjunction error occurs when expectation of conjoint conditions (event intersection) exceeds that of individual constituent parts (Tversky and Kahneman, 1982). This is a logical impossibility. The likelihood of event concurrence (A and B) can never exceed that of component parts (A or B alone) because the possibility set of the conjunction comprises the extension of its constituents (Tversky and Kahneman, 1983). Information representativeness (event typicality) and availability (ease of retrieval) often make conjunctions inappropriately appear more probable than individual elements (Tversky and Kahneman, 1982).

To summarize, heuristic-based decision-making generally generates good/reasonable, adaptive outcomes with minimal cognitive effort. However, in certain situations heuristics can result in bias (i.e., partial, inadequate or preferential appreciation of evidence), which produce sub-optimal judgments. In this context, higher levels of paranormal belief are associated with proneness to statistical bias, specifically misperception of chance.

### Schizotypy and anomalous belief

Schizotypy is a complex, multidimensional psychopathological construct (Lenzenweger, 2015). Conceptually, schizotypy facilitates examination of the schizophrenia-spectrum within the general population. The applicability of schizotypy to non-clinical samples moderates confounds associated with clinical patients (i.e., symptom severity and general decline in cognitive performance) (Barrantes-Vidal et al., 2015; Lenzenweger, 2015). Correspondingly, schizotypy possesses positive, negative and disorganized dimensions and resembles factorial models of schizophrenia (Bentall et al., 1989; Cicero and Kerns, 2010; Dembinska-Krajewska and Rybakowski, 2014).

The positive (psychotic-like) factor encompasses perceptual oddities (i.e., illusions to hallucinations), disruptions in the content of thought (odd beliefs and magical ideation through to delusions), and suspiciousness and paranoia. The negative (deficit) factor references decreased emotional affect (e.g., anhedonia, flattened affect, and disinterest in others and the world). Finally, disorganization includes disruption to thought (organization and expression) and behavior, ranging from mild disturbances to formal thought disorder and grossly disorganized actions. Whilst, there is debate about the appropriateness of this widely published three-factor model, positive and negative schizotypy represent the most robust, consistently replicated dimensions (Kwapil et al., 2012).

Regarding the factorial structure of schizotypy, several theorists propose alternative models. For example, four-factor models comprising cognitive-perceptual, paranoid, negative, and disorganized (Compton et al., 2009) and unusual experiences, cognitive disorganization, introvertive anhedonia and impulsive non-conformity (Mason et al., 2005). The present paper, by virtue of the measure of schizotypy employed (O-Life brief) adopted the model outlined by Mason et al. (2005).

At a theoretical level, conceptualization of schizotypy varies as a function of a researcher's theoretical position on the mental health-mental illness continuum (i.e., quasi-dimensional, dimensional and fully dimensional). The current study assumed the individual differences approach (Claridge and Beech, 1995), which views schizotypy as a personality dimension. This perspective locates respondents on a continuum between relative psychological health and schizophrenia (psychosis) (Barrantes-Vidal et al., 2015). Explicitly, the model delineates schizotypy as a “tendency for characteristics of the psychotic states to be found, in mild degree, among healthy people” (Claridge, 1997, p. 31). This delimitation acknowledges that high levels of schizotypy may exist within non-clinical populations, without developing into spectrum symptoms (Dembinska-Krajewska and Rybakowski, 2014). Furthermore, this paper extended the work of Dagnall et al. (2016b), who also employed the personality perspective.

Specifically, Dagnall et al. (2016b) examined relationships between schizotypy, belief in the paranormal and proneness to heuristic bias. They found that the Unusual Experience (UE) subscale of the Oxford-Liverpool Inventory of Feelings and Experience (O-LIFE scale brief) predicted propensity to both perception of randomness and conjunction fallacy. In comparison, belief in the paranormal was more strongly associated with perception of randomness. Overall, there was a stronger relationship between belief in the paranormal (vs. UE) and statistical bias; level of paranormal belief mediated the strength of association between UE and perception of randomness, but not between UE and conjunction fallacy. Problem type (standard vs. paranormal context) influenced the relationship between UE and statistical bias. Framing strengthened the association between UE and conjunction fallacy.

These results supported Williams and Irwin's (1991) supposition that belief in the paranormal provides a framework for structuring cognitive-perceptual factors associated with positive schizotypy. Findings concurred also with the observation that higher levels of schizotypy and belief in the paranormal were associated with the tendency to perceive unrelated events as connected (causally related) (Dagnall et al., 2016b). Furthermore, Williams and Irwin (1991) reported that schizotypes differed from believers in the paranormal and resembled schizophrenics in terms of cognitive style. Believers conveyed a cognitive style reliant on notions of personal responsibility, while schizotypes emphasized the role of randomness.

Research on schizotypy provides a framework for theorizing about individual differences in cognitive-perceptual style. This extends to anomalous beliefs and the deployment of heuristics strategies. Explicitly, the notion that schizotypal characteristics relate directly to anomalous beliefs and, via the anomalous beliefs-heuristic bias relationship, have an indirect effect on proneness to heuristic bias.

### Belief in the paranormal

Numerous studies have investigated the relationship between propensity to probabilistic reasoning bias and belief in the paranormal. In a widely cited paper, Dagnall et al. (2007) examined whether belief in the paranormal linked to a general weakness in probabilistic reasoning, or was explainable via specific biases. They achieved this by asking participants to complete the Revised Paranormal Belief Scale (RPBS) (Tobacyk and Milford, 1983) alongside probabilistic problems indexing perception of randomness, appreciation of base rate information, susceptibility to conjunction fallacy and derivation of expected value. Analysis revealed that perception of randomness predicted paranormal belief. Median split comparisons supported this finding; participants high (vs. low) in paranormal belief performed less well.

The findings stimulated debate and additional work. Particularly, Rogers et al. (2009, 2011) contended that methodological issues within the Dagnall et al. (2007) study undermined the importance of conjunction error. Using the Scenario Judgments Questionnaire, a newly developed measure, Rogers et al. (2009) observed that believers (vs. non-believers) made more conjunction errors. Additionally, they reported a context effect, whereby standard (vs. paranormal) problems produced more errors. In a follow-up study, Rogers et al. (2011) replicated the finding that believers (vs. non-believers) produced more conjunction errors, but failed to reproduce the previously observed context effect.

Noting the weak, inconsistent nature of conjunction effects and addressing the methodological concerns raised by Rogers et al. (2009, 2011), Dagnall et al. (2014) performed a re-evaluation of the Dagnall et al. (2007) study. Findings supported the original study, perception of randomness best predicted level of paranormal belief. Additionally, conjunction error was weakly associated with only traditional paranormal belief (TPB). TPB is a RPBS dimension related to individual control of social and cultural factors (Goode, 2000). Dagnall et al. (2014) also observed a context effect. Paranormal framed problems proved easier to solve (vs. standard problems), however, this advantage declined within believers.

Dagnall et al. (2016a) extended this paradigm via the addition of paranormal framed perception of randomness problems. Outcomes reinforced those of Dagnall et al. (2014). Explicitly, perception of randomness (vs. susceptibility to conjunction fallacy) was more strongly associated with belief in the paranormal. Conjunction error correlated only with TPB. Further analysis revealed that shared variance with perception of randomness tasks explained the relationship between conjunction and belief in the paranormal. In the context of paranormal belief, proneness to conjunction represented a specific instance of misperception of randomness (Rogers, 2014; Dagnall et al., 2016a). Concerning context effects, results mirrored the previous findings by Dagnall et al. (2014); paranormal framed problems were easier to solve, and this advantage declined as a function of belief in the paranormal.

The observation that believers in the paranormal demonstrate poorer appreciation of randomness was consistent with several previous studies. Notably, Bressan (2002) reported that unusual coincidences (co-occurring random events) were often considered paranormal (causally related) because of the failure to appreciate the probability of chance occurrence. Brugger (1997) explained this in terms of a lower threshold of causal attribution, believers (vs. disbelievers) require less objective evidence in order to establish cause and effect relationships between proximal or attendant events. Indeed, believers are prone to detecting meaning within visual noise patterns (Blackmore and Moore, 1994).

Recent work has qualified the conditions under which conjunction error best relates to belief in the paranormal. Explicitly, believers in the paranormal are prone to endorsing conjunctions when preceding information inductively confirms the constituent (Rogers et al., 2016). Additionally, Prike et al. (2017) found that the relationship between anomalistic belief and conjunction error occurred only for beliefs about having experienced anomalistic phenomena (vs. theoretical anomalistic beliefs).

To the extent that belief in the paranormal is relatively stable, the tendency to endorse such beliefs represents an individual difference. This expresses as variances on cognitive-perceptual factors. Particularly, belief in the paranormal is concomitant with both higher levels of schizotypy and the sub-optimal use of cognitive shortcuts (heuristics).

### The present study

Schizotypy and belief in the paranormal are associated with susceptibility to heuristic bias. This relationship appears to arise from the tendency to connect unrelated events causally (presumably via cognitive-perceptual factors related to odd beliefs and magical thinking) and is linked with specific decrements linked to appreciation of statistical bias (i.e., perception of randomness and conjunction). However, construct overlap between schizotypy and belief in the paranormal make it difficult to determine the relative degree to which each factor contributes to this deficit.

Explicitly, the Schizotypal Personality Disorder diagnostic criteria indexes odd beliefs or magical thinking and unusual perceptual experiences (Hergovich et al., 2008). Odd beliefs or magical thinking are directly relevant to belief in the paranormal as evidenced by specific reference to superstitiousness, belief in clairvoyance, telepathy, or “sixth sense.” The association with unusual experiences, however, is less direct because unusual cognitions and perceptions are not necessary precursors of belief in the paranormal. This link requires an additional attributional phase, which ascribes paranormal causation to unusual experiences (Irwin et al., 2013).

Recognizing this two-tier process, whereby perception of a paranormal experience derives from both detection and labeling of potential anomalies, Irwin et al. (2013) developed the Survey of Anomalous Experiences. This yields two scores, an index of proneness to anomalous experiences (PAE) and a measure of proneness to paranormal attributions (PPA). Irwin et al. (2013) found that the correlation between the unusual experiences (UE) subscale of the O-LIFE and PPA was in the weak to moderate range; adjusting the UE to control for potentially, confounding transpersonal experiences had little effect on this relationship. This finding was consistent with previous studies reporting a moderate positive correlation between schizotypy and belief in the paranormal (Hergovich et al., 2008; Dagnall et al., 2010; Darwin et al., 2011). Furthermore, Hergovich et al. (2008) reported that schizotypy predicted core aspects of belief in the paranormal (i.e., psi, precognition, spiritualism and witchcraft).

Whilst numerous studies document the correlation between schizotypy and belief in the paranormal, the relationship is only moderate. Consequently, associations with related beliefs vary across belief types. For instance, Hergovich et al. (2008) found that schizotypy was a predictor of core components of paranormal belief (i.e., belief in precognition, psi, witchcraft, and spiritualism), whereas belief in traditional religious contents, superstitious thoughts and belief in the existence of extraordinary life forms were better predicted by belief in the paranormal.

Although previous research has consistently established a link between schizotypy and paranormal belief, studies examining this relationship have typically conceptualized these as separate yet related dimensions. For instance, analysis uses correlations, regressions, or path models to examine how the factors relate to probabilistic reasoning performance (e.g., Dagnall et al., 2016b). This reflects a variable-centered approach, which fails to account for the way these dimensions relate within individuals. Some research has carried out cluster analysis to measure within-participant variation in schizotypy, revealing four subtypes (“low schizotypes,” “high schizotypes,” “negative schizotypes,” and “positive/happy schizotypes”) (Loughland and Williams, 1997). In addition, Hori et al. (2014) used latent profile analysis (LPA) to assess homogeneous groups based on schizotypy, temperament and character, also revealing a “positive/adaptive” group, a “negative/maladaptive” group, and a “low schizotypy/adaptive” group. However, research has not examined whether combined profiles of schizotypy and paranormal belief exist. This is surprising given the evidence supporting conceptual overlap and the observation of shared characteristics (odd beliefs and unusual perceptual experiences) (Hergovich et al., 2008).

Using a variable-centered approach assumes that the results from any analysis is an estimate of the relationships among the distinct variables averaged across the whole population, also expected to be homogeneous (Orri et al., 2017). For example, a variable-centered analysis could reveal that levels of paranormal beliefs are high and levels of schizotypy are low in a given sample. Contrastingly, a person-centered approach in this same scenario could identify two qualitatively distinct groups of individuals: one where people possess high paranormal belief and low schizotypy, and another where people possess high paranormal belief and high schizotypy. Person-centered approaches assume populations are heterogeneous, and in relation to paranormal belief and schizotypy could reveal a series of subgroups (i.e., latent profiles) not identified by variable-centered approaches.

Previous research on heuristic bias has examined relationships with schizotypy and paranormal belief independently. Recognizing the potential limitations of this traditional individual differences approach, the present paper adopted a person or case centered perspective. This, via LPA, assessed the conjoint contribution of schizotypy and paranormal belief to statistical bias. Explicitly, LPA identified naturally occurring classes (case profiles) that shared similar cognitive-perceptual characteristics. Accordingly, class membership provided a method for assessing whether the interaction between paranormal belief and schizotypy influenced performance on probabilistic reasoning tasks. From a variable-centered approach, this was not possible. A further advantage of LPA (a specific instance of a finite mixture model) was that subgroups were determined via rigorous empirical tests.

To our knowledge, no study has previously examined latent profiles related to schizotypy and paranormal belief. The advantage of this approach was that it facilitated examination of the degree to which presence and absence of schizotypy and belief in the paranormal influenced probabilistic reasoning performance. Since this was a novel approach, the researchers did not specify the number of latent profiles in advance. Based on existing evidence, the researchers anticipated that probabilistic reasoning task completion would be lower as a function of higher levels of schizotypy and belief in the paranormal (e.g., Dagnall et al., 2017).

## Materials and methods

### Participants

A convenience sample of 725 participants (194 men, 27%; 531 women, 73%) took part in this study. They were recruited via emails to university staff/students and local stakeholders (business, leisure and vocational). The sample mean age was 25.50 (*SD* = 9.40) with a range of 18–64 years (male age, *M* = 27.55, *SD* = 10.30, range of 18–41 years; female age, *M* = 24.75, *SD* = 8.94, range of 18–64 years). Within the sample, 530 (73%) were university students studying at a large post-92 UK university (former polytechnic awarded university status through the Further and Higher Education Act 1992); 195 (27%) were non-students. Participants had to be at least 18 years of age to take part. Prior to participation, a question asked whether participants had previously studied or taken part in studies investigating heuristic bias. If participants endorsed the question, participation discontinued.

### Measures

#### The oxford-liverpool inventory of feelings and experiences (O-LIFE)

The O-LIFE brief (Mason et al., 2005) is an abbreviated form of the original 104-item O-LIFE measure (Mason et al., 1995). The shortened scale comprises 43 items. These represent four dimensions: Unusual Experiences (UE) (12 items), Cognitive Disorganization (CD) (11 items), Introvertive Anhedonia (IA) (10 items) and Impulsive Non-conformity (IN) (10 items). The UE sub-scale taps positive schizotypy (perceptual aberrations, magical thinking and hallucinations). The CD sub-scale measures thought disorder and other disorganized aspects of psychosis, specifically social anxiety, poor attention/concentration and decision-making. The IA sub-scale indexes negative schizotypy (schizoid temperament), particularly avoidance of intimacy and lack of enjoyment from social and physical sources of pleasure. The IN sub-scale measures lack of self-control (i.e., impulsive, anti-social, and eccentric forms of behavior). Collectively, the O-LIFE brief assesses schizotypal personality traits in non-clinical individuals. The O-LIFE possesses sound psychometric qualities, specifically high internal consistency (Mason et al., 1995) and good test–retest reliability (Burch et al., 1998). Since development, studies across psychology related-disciplines have used the O-Life (Mason and Claridge, 2006).

In this study, the internal consistency of the O-Life brief total scale, assessed via Cronbach's alpha (α), was very good (α = 0.87). Internal consistency was also good for UE (α = 0.76) and CD (α = 0.81). Alpha reliability for IA (α = 0.65) and IN (α = 0.63), however, suggested only moderate strength of association among items. Values across dimensions were consistent with those reported by Mason et al. (2005). Concerning Cronbach's alpha, when the degree of measurement error in psychological/social science is considered, 0.6 is an acceptable level (Nunnally, 1978).

#### Revised paranormal belief scale (RPBS)

The RPBS (Tobacyk and Milford, 1983) assesses belief in the paranormal and is the most widely used self-report measure of its type (Goulding and Parker, 2001). The scale contains 26-items, which index seven belief dimensions: spiritualism, witchcraft, precognition, superstition, psi, traditional religious beliefs and extraordinary life forms. Items appear as statements (e.g., “black magic really exists”) and respondents rate each item on a seven-point Likert scale ranging from 1 (strongly disagree) to 7 (strongly agree). Whilst, sub-scale dimensionality remains contested, the RPBS overall demonstrates satisfactory reliability (Cardeña et al., 2015). Acknowledging these issues, Lange et al. (2000) conducted a purification of the RPBS using Rasch scaling and top-down purification. From this process, two psychometrically superior factors emerged, New Age Philosophy (NAP) (11-items evaluating belief in psi and survival of bodily death) and Traditional Paranormal Belief (TPB) (5-items assessing belief in concepts, such as the devil, witchcraft, heaven and hell) (Cardeña et al., 2015). These factors corrected for differential item functioning and accordingly were free from age or gender bias. These dimensions reflect the functions of beliefs. NAP provides control over external events, whereas TPB regulates social/cultural factors (Goode, 2000). The Rasch scaling procedure requires that participant responses are recoded (0–6) (Lange et al., 2000), hence adjusted overall scores range from 0 to 156, with higher scores representing belief in the paranormal. Scores on TPB range from 11.16 to 43.24, and for NAP range from 6.85 to 47.72 (Andrich, 1988). Previous research established that the RPBS possesses adequate validity (Tobacyk, 2004). In this study, internal consistency was very good for the total RPBS scale (α = 0.88) and NAP (α = 0.82), and was good for TPB (α = 0.73).

#### Probabilistic reasoning tasks

Four problem types obtained from Dagnall et al. (2007, 2014) assessed probabilistic reasoning ability (perception of randomness, conjunction fallacy, paranormal perception of randomness and paranormal conjunction fallacy). Arnott's taxonomy of decision biases (1998, 2006) locates judgments of likelihood (perception of randomness and conjunction) within a common statistical bias category.

There were five instances of each problem type, which were organized into four counter-balanced sections. Thus, scores for each problem type ranged from between 0 and 5. Participants responded to each item by indicating which outcome they believed was most probable from the presented range of options. To assist comparisons, raw scores appear alongside proportions; these represent the number of correct responses calculated as a percentage-hit rate. These problems have featured within several previously published studies (Dagnall et al., 2007, 2014, 2016a).

##### Perception of randomness

Problems assessed participants' ability to assess the likelihood of strings/sequences (e.g., “imagine a coin was tossed six times. Which pattern of results do you think is most likely?: (a) HHHHHH, (b) HHHTTT, (c) HTHHTT, (d) all equally likely”).

##### Conjunction fallacy

Conjunction tasks evaluated the ability to recognize that the likelihood of event intersection probability could not exceed that of single (constituent) events (cf. Tversky and Kahneman, 1982, 1983) (e.g., “two football teams (Team A and Team B) are playing in a local derby. What is the most likely outcome of the game?: (a) Team A scores first, (b) Team A scores first and win, (c) Team A scores first and loses, (d) Team A scores first and the game is drawn”).

##### Paranormal perception of randomness

Paranormal perception of randomness items presented judgments about the likelihood of strings/sequences occurrence within a paranormal context. For example, “A famous psychic, with renowned paranormal abilities, has successfully predicted the outcome of the last 6 annually held boat races between two famous English Universities [University A and University B]. This year the psychic predicts University B will win. Which of the following is most likely?: (a) University A will win the event, (b) University B will win the event, (c) University A and University B are both equally as likely to win the event.” These items had the same underlying structure as standard perception of randomness problems.

##### Paranormal conjunction fallacy

Paranormal conjunctions presented each conjunction within a paranormal context. For instance, “Andrew often sits by the telephone at work. Just as he is thinking about his friend, she rings: (a) Elaine rang because Andrew was thinking about her [event intersection], (b) Andrew was thinking about Elaine because she was about to ring [event intersection], (c) Elaine rang [single event]).” Paranormal conjunction fallacy problems possessed the same structure as standard conjunctions (the probability of event intersection could not exceed the likelihood of single (constituent) events.

### Procedure

Prospective participants read the study brief prior to deciding whether to take part in the study. Participants indicated informed consent by ticking a box prior to participation. This protocol ensured that respondents understood the instructions. No personal information was collected other than age, occupational status, preferred gender and general location. Consenting participants received the materials booklet. Study instructions requested that participants answer questions honestly and work through items systematically in their own time. Study booklets comprised three sections: demographic information (completed first), problem tasks, schizotypy and paranormal belief measures. The presentation of schizotypy and paranormal belief measures rotated across participants. Following completion of the materials booklet participants were debriefed.

### Ethics

The researchers obtained ethical approval for the study as part of an unsuccessful research bid. Specifically, the Director of the Research Institute for Health and Social Change (Faculty of Health, Psychology and Social Care) within Manchester Metropolitan University ratified the project (this includes ethical scrutiny and gaining clearance in principal); 01/08/2016. It is also a university condition that the relevant Departmental Head authorizes the project. This is the necessary level of ethical clearance for projects rated as “routine.” Furthermore, members of the Professoriate (or equivalent) peer-review proposals prior to submission. Formal submission to a university ethics panel beyond this process is not an institutional requirement for routine studies.

### Analysis

Analyses used SPSS 23, apart from LPA, which required Mplus version 7 (Muthén and Muthén, 2012). Following initial data screening preliminary analysis computed descriptive statistics. Next, based on O-Life and RPBS scores exploratory LPA determined latent group membership. The first stage in model fit involved evaluating a 1-class model. The number of latent classes in subsequent models increased until the addition of further classes was not justified.

The optimal number of latent classes was determined by considering a range of indices; the Akaike Information Criterion (AIC; Akaike, 1987), the Bayesian Information Criterion (BIC; Schwarz, 1978), the sample-size adjusted BIC (ssaBIC; Sclove, 1987), the Lo-Mendell-Rubin-adjusted likelihood ratio test (LMR-A-LRT; Lo et al., 2001), and a standardized measure of entropy (Ramaswamy et al., 1993). For AIC, BIC, and ssaBIC smaller values indicate better fit. The LMR-A-LRT does not rely on chi-square distribution for the difference in model likelihood values. LMR-A-LRT values normally occur alongside associated *p*-values. Progressive class solutions are computed until a non-significant LMR-A-LRT *p*-value occurs, which indicates a non-significant improvement in fit. Lastly, entropy ranges from 0 to 1, with higher values suggesting a better classification of participants. An entropy value above 0.8 reflects a sound separation of identified classes in relation to the data (Ramaswamy et al., 1993).

Latent class membership acted as a group variable (independent variable) for assessing whether differences existed on probabilistic reasoning task performance (dependent variable). Multivariate analysis of covariance, with possible confounders (defined below) as covariates, then allowed comparison of group performance on probabilistic reasoning tasks. Lastly, *t*-tests examined framing effects (i.e., whether framing probabilistic reasoning tasks in a paranormal context influenced performance).

## Results

### Descriptive statistics

Prior to analysis, data screening removed outliers. Three z-scores were marginally greater than 3.25, these were transformed to the next highest score (Tabachnick and Fidell, 2001). As can be seen from Table 1, all schizotypy-total and sub-factor scores, apart from Introverted Anhedonia (IA), correlated positively with paranormal belief-total and sub-factors (New Age Philosophy, NAP, and Traditional Paranormal Belief, TPB). In addition, probabilistic reasoning performance correlated negatively with schizotypy and paranormal belief scores. The exception to this trend was IA, which did not significantly correlate with perception of randomness (PR) or conjunction fallacy (CF).

**Table 1:** Descriptive statistics and intercorrelations among all study variables.

**Variable**	**Mean**	***SD***	**1**	**2**	**3**	**4**	**5**	**6**	**7**	**8**	**9**	**10**	**11**	**12**
1. O-Life-Total	15.00	7.69		0.79^**^	0.85^**^	0.58^**^	0.78^**^	0.23^**^	0.24^**^	0.23^**^	−0.15^**^	−0.11^*^	−0.25^**^	−0.16^**^
2. UE	3.71	2.70			0.52^**^	0.27^**^	0.54^**^	0.32^**^	0.32^**^	0.34^**^	−0.16^**^	−0.13^*^	−0.33^**^	−0.17^**^
3. CD	5.40	3.16				0.33^**^	0.58^**^	0.17^**^	0.17^**^	0.17^**^	−0.09^*^	−0.09^*^	−0.15^**^	−0.06
4. IA	2.33	2.04					0.28^**^	−0.01	0.01	−0.03	−0.05	−0.03	−0.14^**^	−0.11^*^
5. IN	3.56	2.16						0.17^**^	0.19^**^	0.18^**^	−0.13^**^	−0.07^*^	−0.15^**^	−0.16^**^
6. RPBS-Total	64.93	31.36							0.88^**^	0.82^**^	−0.24^**^	−0.14^**^	−0.45^**^	−0.37^**^
7. NAP	17.82	13.24								0.75^**^	−0.20^**^	−0.11^*^	−0.45^**^	−0.41^**^
8. TPB	9.28	7.45									−0.31^**^	−0.16^**^	−0.42^**^	−0.35^**^
9. PR	3.76	1.06										0.19^**^	0.23^**^	0.38^**^
10. CF	1.91	1.29											0.26^**^	0.13^**^
11. PCF	4.29	1.09												0.52^**^
12. PPR	4.43	1.13												

*O-Life-Total, Oxford-Liverpool Inventory of Personal Experiences-Total; UE, Unusual Experiences; CD, Cognitive Disorganization; IA, Introverted Anhedonia; IN, Impulsive Non-conformity; RPBS-Total, Revised Paranormal Belief Scale-Total; NAP, New Age Philosophy; TPB, Traditional Paranormal Beliefs; PR, Perception of Randomness; CF, Conjunction Fallacy; PCF, Paranormal Conjunction Fallacy; PPR, Paranormal Perception of Randomness*.

* Indicates p < 0.05;

** *Indicates p < 0.001*.

Previous research reports gender differences in paranormal belief (Tobacyk and Milford, 1983; Irwin, 1993; Dag, 1999). Consideration of gender revealed significant differences in relation to Paranormal Conjunction Fallacy (PCF), IA, Paranormal Belief-Total (RPBS), NAP, and TPB (see Table 2). Men scored higher on solving PR, CF, and PCF probabilistic reasoning tasks and possessed greater IA levels. Women reported significantly greater levels of paranormal belief. Due to these differences, subsequent analyses (LPA and multivariate analysis of covariance) included gender as a covariate.

**Table 2:** Means, standard deviations and *t*-test outcomes for all study variables by gender.

	**Gender**					
	**Men**		**Women**		**Independent** ***t*****-test**	
**Variable**	**Mean**	***SD***	**Mean**	***SD***	***t*(*df* = 723)**	***Sig*.**
PR	3.90	1.04	3.71	1.06	2.09	0.037
CF	2.11	1.59	1.84	1.16	2.12	0.035
PPR	4.52	1.07	4.40	1.15	1.31	0.189
PCF	4.52	0.85	4.21	1.16	3.42	<0.001^**^
O-Life-Total	15.65	8.64	14.76	7.31	1.38	0.166
UE	3.93	2.72	3.63	2.70	1.33	0.184
CD	5.05	3.25	5.52	3.12	−1.77	0.077
IA	2.84	2.43	2.15	1.88	4.04	<0.001^**^
IN	3.82	2.34	3.45	2.09	2.04	0.041
RPBS-Total	54.07	26.40	68.90	32.10	−6.30	<0.001^**^
NAP	13.13	10.93	19.53	13.60	−6.50	<0.001^**^
TPB	6.79	6.31	10.18	7.63	−6.02	<0.001^**^

** *Indicates p < 0.004 (Bonferroni adjustment)*.

### Latent profile analysis

LPA used schizotypy and paranormal belief sub-factor scores, gender was included as a covariate. For the purposes of LPA, RPBS scores were converted. Explicitly, ratings of 0–3 were coded as “0” (indicating uncertainty or disagreement) and ratings of 4–6 were coded as “1” (agreement). This method of recoding for LPA is consistent with previous research using Likert scales (e.g., see Deleuze et al., 2015; Hussain et al., 2015).

An initial comparison of 1-class and 2-class models was undertaken. AIC, BIC and ssaBIC indices suggested superior fit of the 2-class model, and the LMR-A-LRT for the 2-class model indicated significant improvement over the 1-class model (see Table 3). A comparison of 2-class and 3-class solutions revealed that the 3-class solution was superior, due to lower AIC, BIC, ssaBIC statistics, higher entropy (0.86 vs. 0.82), and a significant LMR-A-LRT *p*-value. Next, a 4-class solution designated superior fit in comparison with a 3-class solution, evident from lower AIC, BIC, ssaBIC statistics, higher entropy (0.87 vs. 0.86), and a significant LMR-A-LRT *p*-value. A five-class model indicated a non-significant improvement over the 4-class solution; hence, there was no further consideration of solutions.

**Table 3:** Fit of competing latent profile models.

**Model**	**AIC**	**BIC**	**ssaBIC**	**LMR-A**	**LMR-A** ***p-*value**	**Entropy**
1-class	20,317.18	20,381.39	20,336.93			
2-class	19,548.70	19,649.59	19,579.74	769.86	<0.001	0.82
3-class	19,053.84	19,191.42	19,096.16	501.34	<0.001	0.86
4-class	18,833.46	19,007.73	18,887.07	231.97	0.005	0.87
5-class	18,705.28	18,916.24	18,770.18	141.49	0.200	0.87

*AIC, Akaike Information Criterion; BIC, Bayesian Information Criterion; ssaBIC, sample-size adjusted BIC; LMR-A, Lo-Mendell-Rubin-adjusted likelihood ratio test*.

**Figure 1:**
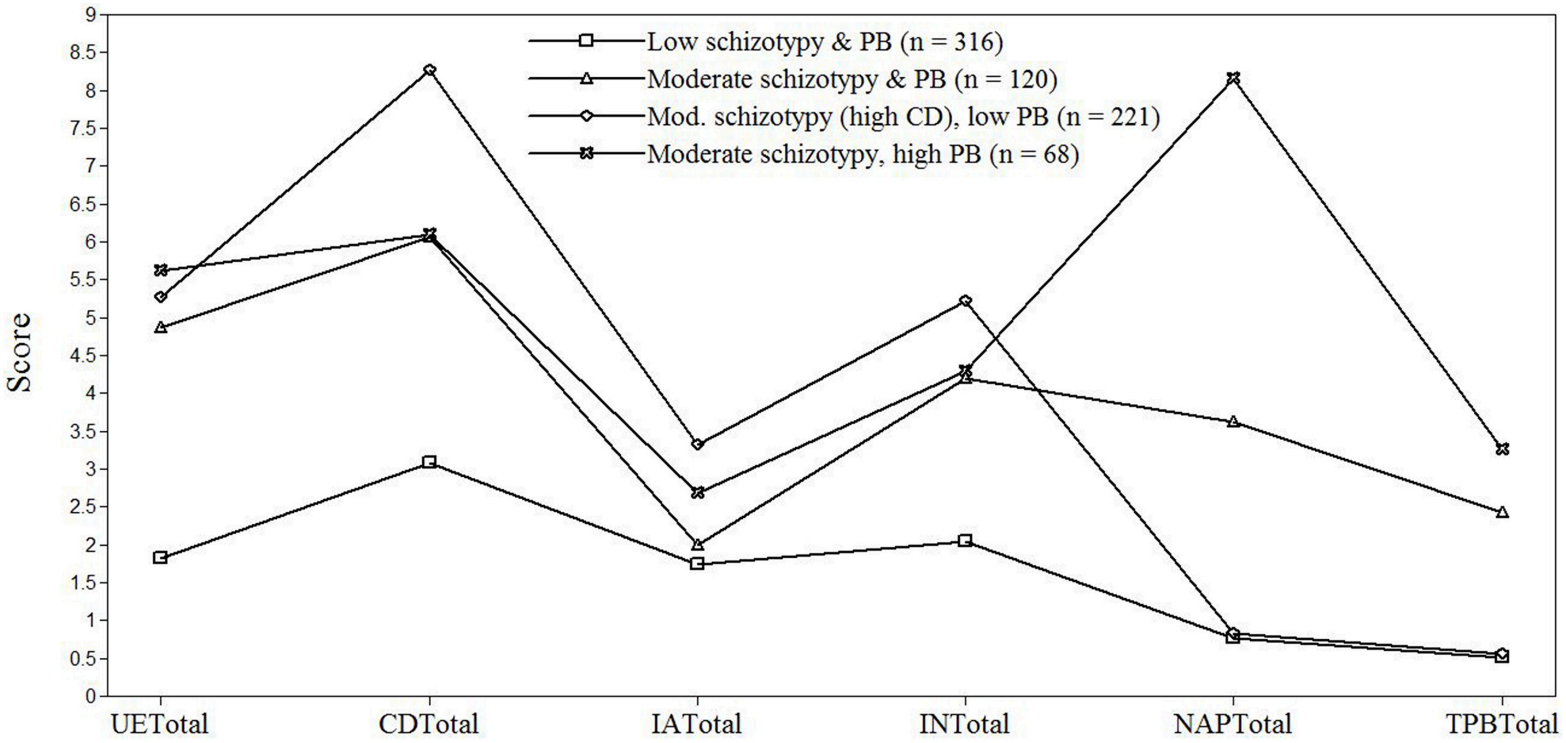
Pattern of mean scores on the Oxford-Liverpool Inventory of Feelings and Experiences (O-Life) and the Revised Paranormal Belief Scale (RPBS) as a function of latent class. PB, Paranormal Belief; UETotal, Unusual Experiences-Total; CDTotal, Cognitive Disorganization-Total; IATotal, Introverted Anhedonia-Total; INTotal, Impulsive Non-conformity-Total; NAPTotal, New Age Philosophy-Total; TPBTotal, Traditional Paranormal Belief-Total.

The 4-class solution represented the best fitting model. In this model (see Figure 1), 43.9% (*n* = 316) of the sample were classified into class 1, and scored low on both schizotypy and paranormal belief. Class 2 represented 18.2% (*n* = 120), and evidenced intermediate scores on schizotypy and paranormal belief. Class 3 comprised 29% (*n* = 221), demonstrating low scores on paranormal belief and intermediate scores on schizotypy apart from Cognitive Disorganization (CD) which reflected a high score. Finally, 8.9% (*n* = 68) comprised class 4, and evidenced intermediate schizotypy scores and high scores on paranormal belief, particularly NAP. Based on these profiles, class 1 represented a “low schizotypy and low paranormal belief” group, class 2 a “moderate schizotypy and moderate paranormal belief” group, class 3 a “moderate schizotypy (high CD) and low paranormal belief” group, and class 4 a “moderate schizotypy and high paranormal belief” group. Average latent class probabilities for most likely latent class membership were 0.95 for class 1, 0.84 for class 2, 0.94 for class 3, and 0.93 for class 4, indicating good overall discrimination.

### Association of latent profiles with probabilistic reasoning task performance

To examine group performance on probabilistic reasoning tasks, multivariate analysis of covariance with gender as a covariate was undertaken (see Table 4). Analysis revealed a significant main effect of group, Pillai's trace = 0.31, *F*_(12,2,157)_ = 20.37, *p* < 0.001, η^2^ = 0.102 (medium effect size). The analysis also reported significant effects of group in relation to each probabilistic reasoning task (perception of randomness, conjunction fallacy, paranormal conjunction fallacy, paranormal perception of randomness). Gender had a non-significant effect on probabilistic reasoning performance, Pillai's trace = 0.02, *F*_(4, 717)_ = 2.37, *p* = 0.051.

**Table 4:** The effects of group (latent profile) on probabilistic reasoning task performance.

	**Dependent Variable**						
	**PR**	**CF**	**PPR**	**PCF**			
	**ANCOVA**					**MANCOVA**	
	***F^*df*^* (*Sig*.; η^2^)**	***F^*df*^* (*Sig*.; η^2^)**	***F^*df*^* (*Sig*.; η^2^)**	***F^*df*^* (*Sig*.; η^2^)**	**Pillai's trace**	***F^*df*^* (*Sig*.)**	**η^2^**
**Variable**							
Group	12.79^3,720^ (<0.001; 0.051)	2.89^3,720^ (0.035; 0.012)	79.04^3,720^ (<0.001; 0.248)	53.01^3,720^ (<0.001; 0.181)	0.31	20.37^12,2157^ (<0.001)	0.102
**Covariate**							
Gender	1.36^1,720^ (0.243; 0.002)	5.16^1,720^ (0.023; 0.007)	3.10^1,720^ (0.079; 0.004)	0.18^1,720^ (0.665; 0.000)	0.02	2.38^4,717^ (0.051)	0.013
	**Pairwise Comparisons (Mean Differences) between Classes**						
**Class contrast**	**Mean diff.** **(*****Sig***.**)**	**Mean diff.** **(*****Sig***.**)**	**Mean diff.** **(*****Sig***.**)**	**Mean diff.** **(*****Sig***.**)**			
Class 1 vs. Class 2	0.49 (<0.001)	0.23 (0.635)	0.91 (<0.001)	0.67 (<0.001)			
Class 1 vs. Class 3	0.13 (0.812)	0.27 (0.106)	0.37 (<0.001)	0.11 (1.0)			
Class 1 vs. Class 4	0.72 (<0.001)	0.38 (0.176)	1.82 (<0.001)	1.62 (<0.001)			
Class 2 vs. Class 3	−0.36 (0.017)	0.04 (1.0)	−0.54 (<0.001)	−0.56 (<0.001)			
Class 2 vs. Class 4	0.23 (0.885)	0.15 (1.0)	0.90 (<0.001)	0.95 (<0.001)			
Class 3 vs. Class 4	0.59 (<0.001)	0.11 (1.0)	1.44 (<0.001)	1.51 (<0.001)			

*PR, Perception of randomness; CF, Conjunction Fallacy; PPR, Paranormal Perception of Randomness; PCF, Paranormal Conjunction Fallacy; Class 1, Low schizotypy and low paranormal belief (n = 316); Class 2, Moderate schizotypy and moderate paranormal belief (n = 120); Class 3, Moderate schizotypy, high CD and low paranormal belief (n = 221); and Class 4, Moderate schizotypy and high paranormal belief (n = 68)*.

*Post-hoc* pairwise comparisons with Bonferroni correction (Table 4) revealed that the “low schizotypy and low paranormal belief” group (*M* = 3.96, *SD* = 0.96) performed significantly better on PR tasks than the “moderate schizotypy and moderate paranormal belief” group (*M* = 3.45, *SD* = 1.07) and the “moderate schizotypy and high paranormal belief” group (*M* = 3.22, *SD* = 1.47). The “moderate schizotypy (high CD) and low paranormal belief” group (*M* = 3.83, *SD* = 0.97) were significantly better at solving PR problems than the “moderate schizotypy and moderate paranormal belief” group (*M* = 3.45, *SD* = 1.07) and the “moderate schizotypy and high paranormal belief” group (*M* = 3.22, *SD* = 1.47). Interestingly, there existed no meaningful differences in performance on CF tasks among the distinct latent profiles.

The “low schizotypy and low paranormal belief” group (*M* = 4.73, *SD* = 0.56) were the most adept at solving PCF problems, followed by the “moderate schizotypy (high CD) and low paranormal belief” group (*M* = 4.37, *SD* = 0.91), and the “moderate schizotypy and moderate paranormal belief” group (*M* = 3.80, *SD* = 1.38). The “moderate schizotypy and high paranormal belief” group (*M* = 2.90, *SD* = 1.46) performed the worst at solving PCF problems.

Similarly, the “low schizotypy and low paranormal belief” group (*M* = 4.73, *SD* = 0.81) were the most proficient at solving PPR problems, followed by the “moderate schizotypy (high CD) and low paranormal belief” group (*M* = 4.62, *SD* = 0.78), and the “moderate schizotypy and moderate paranormal belief” group (*M* = 4.07, *SD* = 1.38). The “moderate schizotypy and high paranormal belief” group (*M* = 3.12, *SD* = 1.70) performed the worst at solving PPR problems.

To examine framing effects, a series of *t*-tests were conducted *post-hoc* (with a Bonferroni adjusted alpha of 0.006) comparing PR and CF task performance with PPR and PCF task performance for each latent profile (see Table 5). A framing effect was present for the “low schizotypy and low paranormal belief” group, evident by the significantly greater scores in task performance for PPR vs. PR and PCF vs. CF. Similarly, for the “moderate schizotypy and moderate paranormal belief” group, PPR performance was significantly greater than PR performance, and PCF performance was significantly greater than CF performance. For the “moderate schizotypy (high CD) and low paranormal belief” group, participants recorded greater performance on PPR vs. PR tasks and on PCF vs. CF tasks. A framing effect was not apparent for the “moderate schizotypy and high paranormal belief” group in relation to perception of randomness, but seemed to occur for conjunction problems.

**Table 5:** Probabilistic reasoning performance as a function of class membership.

	**Probabilistic reasoning type**												**Comparisons**			
	**Perception of randomness (PR)**			**Paranormal perception of randomness (PPR)**			**Conjunction fallacy (CF)**			**Paranormal conjunction fallacy (PCF)**			**PR vs. PPR**		**CF vs. PCF**	
**Class membership**	***M***	***SD***	**Prop. correct (%)**	***M***	***SD***	**Prop. correct (%)**	***M***	**SD**	**Prop. correct (%)**	***M***	***SD***	**Prop. correct (%)**	***t* (*df*)**	***Sig*.**	***t* (*df*)**	***Sig*.**
Class 1	3.96	0.96	79	4.73	0.81	95	2.08	1.43	42	4.73	0.56	95	−11.69 (315)	<0.001^**^	−32.82 (315)	<0.001^**^
Class 2	3.45	1.07	69	4.07	1.38	81	1.82	1.23	36	3.80	1.38	76	−4.64 (119)	<0.001^**^	−13.64 (119)	<0.001^**^
Class 3	3.83	0.97	77	4.62	0.78	92	1.82	1.19	36	4.37	0.91	87	−12.25 (220)	<0.001^**^	−31.08 (220)	<0.001^**^
Class 4	3.22	1.47	64	3.12	1.70	62	1.66	0.97	33	2.90	1.46	58	0.56 (67)	0.578	−6.99 (67)	<0.001^**^
Overall (*N* = 725)	3.80	1.07	72	4.44	1.13	83	1.92	1.30	37	4.30	1.10	79				

Class 1, Low schizotypy and low paranormal belief (n = 316); Class 2, Moderate schizotypy and moderate paranormal belief (n = 120); Class 3, Moderate schizotypy, high CD and low paranormal belief (n = 221); and Class 4, Moderate schizotypy and high paranormal belief (n = 68);

** *Indicates p < 0.006 (Bonferroni adjustment)*.

## Discussion

### General summary

This study assessed the extent to which latent class membership, combining schizotypy and belief in the paranormal, influenced performance on probabilistic reasoning tasks assessing proneness to statistical bias (perception of randomness and conjunction error). Analysis identified four latent profiles (low schizotypy and low belief in the paranormal; moderate schizotypy and moderate belief in the paranormal; moderate schizotypy, high cognitive disorganization and low paranormal belief; and moderate schizotypy and high paranormal belief). These comprised scores ranging from lower to higher scores on schizotypy and belief in the paranormal and reflected the most appropriate variable combinations within the present sample.

### Latent profile analysis (LPA) outcomes

The emergence of low, moderate, and to an extent high schizotypy (i.e., cognitive disorganization; CD) profiles is consistent with previous within-variable analyses (Loughland and Williams, 1997; Hori et al., 2014). A counter-intuitive result concerned class 3 (moderate schizotypy, high CD and low paranormal belief). Specifically, 29% of the sample appeared to possess low paranormal belief, but the highest schizotypy levels. This observation seems to fit with the proposal of Hergovich et al. (2008), who suggested that although conceptual overlap exists, schizotypy might not necessarily involve paranormal belief. Research evidencing a moderate correlation between schizotypy and paranormal belief supports this proposal (Dagnall et al., 2010; Darwin et al., 2011). It is likely, therefore, that a subgroup of individuals exists who are relatively high on schizotypy but do not particularly endorse paranormal beliefs.

The identification of class 3 demonstrated the value of LPA. Pure variable-driven research would not have identified this respondent class. To ensure this cluster represents a reliable class subsequent research should assess further the extent to which the group exists within other samples. Robustly establishing classes across samples will help to confirm cluster cognitive characteristics and validate between class comparisons. Sophisticated appreciation of the cognitive profiles of individuals vis-à-vis those who score high (low) on both traits will produce a nuanced understanding of anomalous belief. Specifically, the degree to which variations in schizotypy effect belief acquisition, organization and processing.

The emergence of a group (class 4) possessing moderate schizotypy and high paranormal belief (particularly New Age Philosophy; NAP) concurs with the findings of Houran et al. (2001). They concluded that NAP involves beliefs relating to psychopathology or an adverse structure of personality. Specifically, NAP incorporates beliefs that are partly dissociative and, therefore, likely to co-occur with schizotypy.

### Probabilistic reasoning performance as a function of LPA class

Class comparisons revealed that performance on probabilistic reasoning tasks varied across groups and as a function of problem type, and these differences remained after controlling for gender. Regarding standard perception of randomness problems, class 1 (low schizotypy and low paranormal belief) and class 3 (moderate schizotypy, high CD and low paranormal belief) performed better than class 2 (moderate schizotypy and moderate paranormal belief) and class 4 (moderate schizotypy and high paranormal belief). There were no significant differences between classes 1 vs. 3 and 2 vs. 4.

These results indicated that low levels of paranormal belief were associated with superior performance on perception of randomness tasks. Level of schizotypy had only a negligible effect on performance. Moderate levels of schizotypy were concomitant only with lower levels of task solution when individuals also possessed high levels of paranormal belief. These results were consistent with Dagnall et al.'s (2016b) conclusion that belief in the paranormal was more disruptive to perception of randomness task solution than schizotypy.

Specifically, Dagnall et al. (2016b) found that belief in the paranormal mediated the association between schizotypy (Unusual Experiences) and perception of randomness. Within the present study, there was a clear difference as a function of level of belief in the paranormal. This finding supported the notion that belief in the paranormal is associated with misperception of randomness (Dagnall et al., 2007, 2014, 2016a,b; Dagnall et al., 2017).

Contrastingly, performance on standard conjunction tasks did not vary significantly as a function of class. In comparison to perception of randomness tasks, overall conjunction scores were lower (72% vs. 37%). Participants were generally less proficient at solving conjunction tasks. Indeed, results indicated that levels of belief in the paranormal and schizotypy had only a minor influence on the ability to solve conjunction fallacy problems. Overall, findings supported preceding work by Dagnall et al. (2007, 2014, 2016a,b), which suggested that conjunction (vs. perception of randomness) tasks are more difficult to solve and less strongly related to level of belief in the paranormal (Dagnall et al., 2017).

Regarding framing, when problems were located within a paranormal context, solution rate increased (perception of randomness, 83% and conjunction fallacy, 79%). In the case of paranormal perception of randomness, outcomes paralleled the pattern of results found for standard perception of randomness tasks. Explicitly, classes 1 and 3 performed better than classes 2 and 4. The major difference was class 4 (moderate schizotypy and high paranormal belief group), who scored lower than class 2 (moderate schizotypy and moderate paranormal belief). This occurred because performance within class 4 failed to increase; solution rates within other classes improved relative to standard versions. Participants with higher levels of paranormal belief found the paranormal perception of randomness problems as difficult to solve as standard problems. Placing perception of randomness problems within a paranormal context generally makes them easier to solve, however, this advantage does not apply to participants high in belief in the paranormal (particularly NAP) and schizotypy.

Performance on paranormal conjunction fallacy problems demonstrated a different pattern of results. Although all participants performed significantly better on paranormal (vs. standard) problems, level of improvement varied as a function of class. Class 1 (low schizotypy and low paranormal belief), scored higher than class 3 (moderate schizotypy, high CD and low paranormal belief), who outperformed class 2 (moderate schizotypy and moderate paranormal belief), and class 4 (moderate schizotypy and high paranormal belief) scored lowest. Probabilistic reasoning task solution varied as a function of the interaction between levels of paranormal belief and schizotypy.

When participants were low on both factors probabilistic reasoning performance was highest, and when participants were relatively high on both factors probabilistic reasoning performance was lowest. Increased levels of schizotypy disrupted the beneficial effects of context for participants low in paranormal belief. This finding concurred with the notion that schizotypy within general populations is only weakly related to propensity to statistical bias (Dagnall et al., 2017). Generally, as with perception of randomness, higher levels of belief in the paranormal were associated with lower levels of improved performance.

It is evident from these results, that participants find standard perception of randomness tasks easier to solve than standard conjunctions. It is also clear that performance on perception of randomness tasks is influenced by levels of paranormal belief (strongly) and schizotypy (weakly), whereas propensity to conjunction error is relatively unaffected by these factors. In the case of both probabilistic reasoning tasks, there was a clear framing effect, whereby paranormal problem types (vs. standard) were generally easier to solve. The only exception was class 4, where performance on paranormal perception of randomness tasks remained unchanged. Finally, level of improvement varied as a function of task type and class membership. Overall, these results provide support for the previous work of Dagnall et al. (2016b, 2017). The addition of LPA provided a more nuanced understanding of how within-individual variation effected probabilistic reasoning performance; an addition that would not be possible to study via a variable-centered approach.

The present study indicated that level of paranormal belief (particularly), and degree of schizotypy influenced proneness to perception of randomness. These findings support the notion that illusory causation, particularly limited appreciation of chance is an important factor associated with belief in the paranormal. This may be because individuals who are higher in schizotypy and endorse paranormal beliefs perceive unrelated events as connected or causally related (Dagnall et al., 2016b). This notion is consistent with the observation that odd beliefs or magical thinking influence perceptions of causation (Hergovich et al., 2008). Belief in the paranormal directly indexes these factors, whilst schizotypy acts as an indirect measure. This view is congruent with the view that belief in the paranormal acts as a worldview or framework in which to interpret odd thinking, unusual perceptions and experiences. The remaining elements of schizotypy as defined by the Schizotypal Personality Disorder diagnostic criteria are less relevant to belief in the paranormal and perception of causation.

### Limitations

A limitation of the present study concerned the relative distributions of belief in the paranormal and schizotypy. Precisely, greater variation existed within the paranormal belief scores in comparison to schizotypy. Whilst schizotypy totals accorded with norms established by Mason et al. (2005), range restriction occurred because respondents came from a general, non-clinical population. Hence, latent profiles only reflected differences between low and moderate schizotypy. Greater performance disruption may be evident in samples when higher levels of schizotypy are present. Considering these factors, the results indicate that level of paranormal belief effects the ability to solve perception of randomness problems and suggest that only high levels of schizotypy influence task solution. To assess this notion further, subsequent research needs to ensure that samples possess similar ranges of paranormal belief and schizotypy. More generally, given sample limitations, the present findings require cautious interpretation. Additionally, the identified latent profiles require corroboration in more diverse samples; the present sample, whilst heterogeneous contained a large percentage of students. This point is especially important given the data-driven, exploratory nature of LPA. Furthermore, consensus regarding the identification of the current classes or others should remain an active field of inquiry.

Performance of LPA requires caution because recoding continuous data to produce meaningful categorical variables can result in substantial information loss (Lanza and Rhoades, 2013). In the present study, emergent classes were conceptually meaningful and statistically distinguishable, however, it is important to guard against reification. Categories identified via LPA represent merely heterogeneity across dimensions included in the model not types of individuals present within the population (Lanza and Rhoades, 2013). Misspecification can occur if LPA identifies too many or too few classes. To reduce the possibility of misspecification future studies could employ cross-validation methods, such as double cross-validation (see Collins et al., 1994) or progressive elaboration (see Donovan and Chung, 2015). These methods provide useful objective tests of model fit and help to establish class stability. Although, it is important to note that cross-validation indicates only the best approximation to the true model (Collins et al., 1994).

Finally, related research indicates that susceptibility to statistical bias varies as a function of cognitive-perceptual factor (e.g., schizotypy, delusional ideation, hallucination proneness; Dagnall et al., 2015) and belief type (e.g., paranormal belief and conspiratorial ideation; Dagnall et al., 2017). Illustratively, propensity to conjunction error correlates more strongly with conspiratorial ideation than belief in the paranormal (Dagnall et al., 2017). In this context, future studies using latent profiles could provide a more sophisticated understanding of interactions between cognitive-perceptual factors, beliefs and propensity to heuristic bias.

## Author contributions

AD and ND: theoretical focus, data analysis and article development; KD: collected data and contributed to the writing process; AP: contributed to the writing process.

### Conflict of interest statement

The authors declare that the research was conducted in the absence of any commercial or financial relationships that could be construed as a potential conflict of interest.

## References

[B1] Akaike H. (1987). Factor analysis and AIC. Psychometrika 52, 317–332. 10.1007/BF02294359

[B2] Andrich D. (1988). Rasch Models for Measurement, Vol. 68. London: Sage.

[B3] Arnott D. (1998). A Taxonomy of Decision Biases. Melbourne, VIC: Monash University.

[B4] Arnott D. (2006). Cognitive biases and decision support systems development: a design science approach. Inform. Syst. J. 16, 55–78. 10.1111/j.1365-2575.2006.00208.x

[B5] Barrantes-Vidal N. Grant P. Kwapil T. R. (2015). The role of schizotypy in the study of the etiology of schizophrenia spectrum disorders. Schizophr. Bull. 41 (Suppl. 2), S408–S416. 10.1093/schbul/sbu191 25810055 PMC4373635

[B6] Bentall R. P. Claridge G. S. Slade P. D. (1989). The multidimensional nature of schizotypal traits: a factor analytic study with normal subjects. Br. J. Clin. Psycholo. 28, 363–375. 10.1111/j.2044-8260.1989.tb00840.x 2605389

[B7] Blackmore S. Moore R. (1994). Seeing things: visual recognition and belief in the paranormal. Eur. J. Parapsychol. 10, 91–103.

[B8] Blackmore S. Trościanko T. (1985). Belief in the paranormal: probability judgements, illusory control, and the ‘chance baseline shift’. Br. J. Psychol. 76, 459–468.

[B9] Bressan P. (2002). The connection between random sequences, everyday coincidences, and belief in the paranormal. Appl. Cogn. Psychol. 16, 17–34. 10.1002/acp.754

[B10] Brugger P. (1997). Variables that influence the generation of random sequences: an update. Percept. Mot. Skills 84, 627–661. 10.2466/pms.1997.84.2.627 9106859

[B11] Burch G. S. J. Steel C. Hemsley D. R. (1998). Oxford—Liverpool inventory of feelings and experiences: reliability in an experimental population. Br. J. Clin. Psychol. 37, 107–108. 10.1111/j.2044-8260.1998.tb01284.x 9547965

[B12] Cardeña E. Palmer J. Marcusson-Clavertz D. (eds.). (2015). Parapsychology: A Handbook for the 21st Century. Jefferson, NC: McFarland.

[B13] Cicero D. C. Kerns J. G. (2010). Multidimensional factor structure of positive schizotypy. J. Pers. Disord. 24, 327–343. 10.1521/pedi.2010.24.3.327 20545498

[B14] Claridge G. (1997). Schizotypy. Implications for Illness and Health. New York, NY: Oxford University Press.

[B15] Claridge G.S. Beech A. (1995). Fully and quasi-dimensional constructions of schizotypy, in Schizotypal Personality, eds Raine A. Lencz T. (New York, NY: Cambridge), 192–216.

[B16] Collins L. M. Graham J. W. Long J. D. Hansen W. B. (1994). Crossvalidation of latent class models of early substance use onset. Multiv. Behav. Res. 29, 165–183. 10.1207/s15327906mbr2902_3 26745026

[B17] Compton M. T. Goulding S. M. Bakeman R. McClure-Tone E. B. (2009). Confirmation of a four-factor structure of the Schizotypal Personality Questionnaire among undergraduate students. Schizophr. Res. 111, 46–52. 10.1016/j.schres.2009.02.012 19278834

[B18] Costello F. Mathison T. (2014). On fallacies and normative reasoning: when people's judgements follow probability theory in Proceedings of the Cognitive Science Society (Quebec City), 361–366.

[B19] Dag I. (1999). The relationships among paranormal beliefs, locus of control and psychopathology in a Turkish college sample. Pers. Individ. Dif. 26, 723–737. 10.1016/S0191-8869(98)00184-6

[B20] Dagnall N. Denovan A. Drinkwater K. Parker A. Clough P. (2016b). Toward a better understanding of the relationship between belief in the paranormal and statistical bias: the potential role of schizotypy. Front. Psychol. 7:1045. 10.3389/fpsyg.2016.01045 27471481 PMC4943933

[B21] Dagnall N. Denovan A. Drinkwater K. Parker A. Clough P. J. (2017). Urban legends and paranormal beliefs: the role of reality testing and schizotypy. Front. Psychol. 8:942. 10.3389/fpsyg.2017.00942 28642726 PMC5463090

[B22] Dagnall N. Drinkwater K. Denovan A. Parker A. Rowley K. (2016a). Misperception of chance, conjunction, framing effects and belief in the paranormal: a further evaluation. Appl. Cogn. Psychol. 30, 409–419. 10.1002/acp.3217

[B23] Dagnall N. Drinkwater K. Parker A. Denovan A. Parton M. (2015). Conspiracy theory and cognitive style: a worldview. Front. Psychol. 6:206. 10.3389/fpsyg.2015.00206 25762969 PMC4340140

[B24] Dagnall N. Drinkwater K. Parker A. Rowley K. (2014). Misperception of chance, conjunction, belief in the paranormal and reality testing: a reappraisal. Appl. Cogn. Psychol. 28, 711–719. 10.1002/acp.3057

[B25] Dagnall N. Munley G. Parker A. Drinkwater K. (2010). Paranormal belief, schizotypy, and transliminality. J. Parapsychol. 74, 117–141.

[B26] Dagnall N. Parker A. Munley G. (2007). Paranormal belief and reasoning. Pers. Individ. Dif. 43, 1406–1415. 10.1016/j.paid.2007.04.017

[B27] Darwin H. Neave N. Holmes J. (2011). Belief in conspiracy theories. The role of paranormal belief, paranoid ideation and schizotypy. Pers. Indiv. Diff. 50, 1289–1293. 10.1016/j.paid.2011.02.027

[B28] Deleuze J. Rochat L. Romo L. Van der Linden M. Achab S. Thorens G. (2015). Prevalence and characteristics of addictive behaviors in a community sample: a latent class analysis. Addict. Behav. Rep. 1, 49–56. 10.1016/j.abrep.2015.04.001 PMC5845955 29531979

[B29] Dembinska-Krajewska D. Rybakowski J. (2014). The Oxford-Liverpool Inventory of Feelings and Experiences (O-LIFE) schizotypy scale in psychiatry. Arch. Psychiatr. Psychother. 2, 15–22. 10.12740/APP/26780

[B30] Donovan J. E. Chung T. (2015). Progressive elaboration and cross-validation of a latent class typology of adolescent alcohol involvement in a national sample. J. Stud. Alcohol Drugs 76, 419–429. 10.15288/jsad.2015.76.419 25978828 PMC4440300

[B31] Eckblad M. Chapman L. J. (1983). Magical ideation as an indicator of schizotypy. J. Consult. Clin. Psychol. 51, 215–225. 10.1037/0022-006X.51.2.215 6841765

[B32] Gigerenzer G. Gaissmaier W. (2011). Heuristic decision making. Annu. Rev. Psychol. 62, 451–482. 10.1146/annurev-psych-120709-145346 21126183

[B33] Goode E. (2000). Paranormal Beliefs: A sociological Introduction. Prospect Heights, IL: Waveland Press.

[B34] Goulding A. Parker A. (2001). Finding psi in the paranormal: psychometric measures used in research on paranormal beliefs/experiences and in research on psi-ability. Eur. J. Parapsychol. 16, 73–101.

[B35] Hergovich A. Schott R. Arendasy M. (2008). On the relationship between paranormal belief and schizotypy among adolescents. Pers. Individ. Dif. 45, 119–125. 10.1016/j.paid.2008.03.005

[B36] Hori H. Teraishi T. Sasayama D. Matsuo J. Kinoshita Y. Ota M.. (2014). A latent profile analysis of schizotypy, temperament and character in a nonclinical population: association with neurocognition. J. Psychiatr. Res. 48, 56–64. 10.1016/j.jpsychires.2013.10.006 24183242

[B37] Houran J. Irwin H. J. Lange R. (2001). Clinical relevance of the two-factor rasch version of the revised paranormal belief scale. Pers. Individ. Dif. 31, 371–382. 10.1016/S0191-8869(00)00143-4

[B38] Hussain Z. Williams G. A. Griffiths M. D. (2015). An exploratory study of the association between online gaming addiction and enjoyment motivations for playing massively multiplayer online role-playing games. Comput. Human Behav. 50, 221–230. 10.1016/j.chb.2015.03.075

[B39] Irwin H. J. (1993). Belief in the paranormal: a review of the empirical literature. J. Am. Soc. Psychic. Res. 87, 1–39.

[B40] Irwin H. J. Dagnall N. Drinkwater K. (2013). Parapsychological experience as anomalous experience plus paranormal attribution: a questionnaire based on a new approach to measurement. J. Parapsychol. 77, 39–53.

[B41] Kahneman D. Tversky A. (1973). On the psychology of prediction. Psychol. Rev., 80, 237–251. 10.1037/h0034747

[B42] Kwapil T. R. Brown L. H. Silvia P. J. Myin-Germeys I. Barrantes-Vidal N. (2012). The expression of positive and negative schizotypy in daily life: an experience sampling study. Psychol. Med. 42, 2555–2566. 10.1017/S0033291712000827 22716971 PMC12815227

[B43] Lange R. Irwin H. J. Houran J. (2000). Top-down purification of Tobacyk's revised paranormal belief scale. Pers. Individ. Dif. 29, 131–156. 10.1016/S0191-8869(99)00183-X

[B44] Lanza S. T. Rhoades B. L. (2013). Latent class analysis: an alternative perspective on subgroup analysis in prevention and treatment. Prevent. Sci. 14, 157–168. 10.1007/s11121-011-0201-1 21318625 PMC3173585

[B45] Lenzenweger M. F. (2015). Thinking clearly about schizotypy: hewing to the schizophrenia liability core, considering interesting tangents, and avoiding conceptual quicksand. Schizophr. Bull. 41(Suppl. 2), S483–S491. 10.1093/schbul/sbu184 25810061 PMC4373631

[B46] Lo Y. Mendell N. R. Rubin D. B. (2001). Testing the number of components in a normal mixture. Biometrika 88, 767–778. 10.1093/biomet/88.3.767

[B47] Loughland C. M. Williams J. M. (1997). A cluster analytic study of schizotypal trait dimensions. Pers. Individ. Dif. 23, 877–883. 10.1016/S0191-8869(97)00086-X

[B48] Mason O. Claridge G. (2006). The Oxford-Liverpool Inventory of Feelings and Experiences (O-LIFE): further description and extended norms. Schizophr. Res. 82, 203–211. 10.1016/j.schres.2005.12.845 16417985

[B49] Mason O. Claridge G. Jackson M. (1995). New scales for the assessment of schizotypy. Pers. Individ. Dif. 18, 7–13. 10.1016/0191-8869(94)00132-C

[B50] Mason O. Linney Y. Claridge G. (2005). Short scales for measuring schizotypy. Schizophr. Res. 78, 293–296. 10.1016/j.schres.2005.06.020 16054803

[B51] Muthén L. K. Muthén B. O. (2012). Mplus User's Guide, 7th Edn. Los Angeles, CA: Muthén and Muthén.

[B52] Nunnally J. C. (1978). Psychometric Theory, 2nd Edn. New York, NY: McGraw-Hill.

[B53] Orri M. Pingault J. B. Rouquette A. Lalanne C. Falissard B. Herba C.. (2017). Identifying affective personality profiles: a latent profile analysis of the Affective Neuroscience Personality Scales. Sci. Rep. 7, 45–48. 10.1038/s41598-017-04738-x 28674393 PMC5495783

[B54] Prike T. Arnold M. M. Williamson P. (2017). Psychics, aliens, or experience? Using the Anomalistic Belief Scale to examine the relationship between type of belief and probabilistic reasoning. Conscious. Cogn. 53, 151–164. 10.1016/j.concog.2017.06.003 28683360

[B55] Ramaswamy V. DeSarbo W. S. Reibstein D. J. Robinson W. T. (1993). An empirical pooling approach for estimating marketing mix elasticities with PIMS data. Market. Sci. 12, 103–124. 10.1287/mksc.12.1.103

[B56] Rogers P. (2014). Paranormal believers' proneness to probabilistic reasoning biases, in Aberrant Beliefs and Reasoning, ed Galbraith N. D. (Hove: Psychology Press), 114–131.

[B57] Rogers P. Davis T. Fisk J. E. (2009). Paranormal belief and susceptibility to the conjunction fallacy. Appl. Cogn. Psychol. 23, 524–542. 10.1002/acp.1472

[B58] Rogers P. Fisk J. E. Lowrie E. (2016). Paranormal believers' susceptibility to confirmatory versus disconfirmatory conjunctions. Appl. Cogn. Psychol. 30, 628–634. 10.1002/acp.3222

[B59] Rogers P. Fisk J. E. Wiltshire D. (2011). Paranormal belief and the conjunction fallacy: controlling for temporal relatedness and potential surprise differentials in component events. Appl. Cogn. Psychol. 25, 692–702. 10.1002/acp.1732

[B60] Schwarz G. (1978). Estimating the dimension of a model. Ann. Stat. 6, 461–464. 10.1214/aos/1176344136

[B61] Sclove S. L. (1987). Application of model-selection criteria to some problems in multivariate analysis. Psychometrika 52, 333–343. 10.1007/BF02294360

[B62] Shah A. K. Oppenheimer D. M. (2008). Heuristics made easy: an effort-reduction framework. Psychol. Bull. 134, 207–222. 10.1037/0033-2909.134.2.207 18298269

[B63] Tabachnick B. G. Fidell L. S. (2001). Using Multivariate Statistics, 4th Edn. Boston, MA: Allyn and Bacon.

[B64] Thalbourne M. A. French C. C. (1995). Paranormal belief, manic-depressiveness, and magical ideation: a replication. Pers. Individ. Dif. 18, 291–292. 10.1016/0191-8869(94)00146-J

[B65] Tobacyk J. J. (2004). A revised paranormal belief scale. Int. J. Transp. Stud. 23, 94–99.

[B66] Tobacyk J. J. Milford G. (1983). Belief in paranormal phenomena: assessment instrument development and implications for personality functioning. J. Pers. Soc. Psychol. 44, 1029–1037. 10.1037/0022-3514.44.5.1029

[B67] Tversky A. Kahneman D. (1974). Judgment under uncertainty: heuristics and biases. Science 185, 1124–1130. 10.1126/science.185.4157.1124 17835457

[B68] Tversky A. Kahneman D. (1982). Evidential impact of base rates, in Judgement under Uncertainty: Heuristics and Biases, eds Kahneman D. Slovic P. Tversky A. (New York, NY: Cambridge University Press), 153–160.

[B69] Tversky A. Kahneman D. (1983). Extensional versus intuitive reasoning: the conjunction fallacy in probability judgement. Psychol. Rev. 90, 293–315. 10.1037/0033-295X.90.4.293 PMC4658440 26635712

[B70] Tversky A. Kahneman D. (1993). Probabilistic reasoning, in Readings in Philosophy and Cognitive Science, ed Goldman A. I. (Cambridge, MA: MIT Press), 43–68.

[B71] Williams L. M. Irwin H. J. (1991). A study of paranormal belief, magical ideation as an index of schizotypy and cognitive style. Pers. Individ. Dif. 12, 1339–1348. 10.1016/0191-8869(91)90210-3

